# EP32 Gastroparesis in Sjögren’s syndrome

**DOI:** 10.1093/rap/rkaa052.031

**Published:** 2020-11-03

**Authors:** Vera Saulite, Poonam Sharma

**Affiliations:** North West Anglia NHS Foundation Trust, Peterborough, United Kingdom

## Abstract

**Case report - Introduction:**

We present a case of a young woman with long-standing history of difficulty swallowing and progressively worsening abdominal pain who was later found to have weakly positive ANA and microcystic changes in submandibular glands bilaterally. Barium swallow demonstrated gastroparesis and the diagnosis of primary Sjögren’s syndrome (pSS) was made. There is conflicting evidence in the literature on the prevalence of gastroparesis in pSS population. The case is remarkable because abdominal symptoms and problems with swallowing rather than sicca syndrome prompted investigations and later confirmed the diagnosis.

**Case report - Case description:**

A nineteen-year old woman had long-standing issues with dry throat, problems with swallowing and sensation of phlegm in the throat, which she felt had started after she swallowed a bone. Provisional diagnoses of globus pharyngeus and chronic throat clearing were made by ENT team. She had trials of sodium bicarbonate, Gaviscon and PPI. She then had a neck USS to exclude goitre and was found to have microcystic changes in submandibular salivary glands bilaterally. This prompted referral to Rheumatology clinic.

She reported post-prandial abdominal pain with bloating and early satiety. She had transient pruritic rashes. There was no history of joint pains or swelling. She had dry eyes. There was no other history suggestive of connective tissue disorder. Her past medical history included tonsillectomy and iron deficiency anaemia secondary to heavy menses. Her family history was unremarkable.

Examination did not demonstrate synovitis or rashes. Her cardiovascular and abdominal examination was normal. Her blood tests showed negative anticardiolipin antibody, negative ds-DNA, negative MPO and PR3, negative ANA by HEp2 and negative rheumatoid factor. She had a weakly positive smooth muscle antibody and a positive ANA by CIA which was negative by HEp2 and therefore thought to be of doubtful clinical significance. She had normal complement, creatine kinase and CRP, and a low ferritin with low haemoglobin, in keeping with known iron-deficiency anaemia.

In view of iron deficiency and low vitamin D levels coeliac screen was performed which was negative. Her chest x-ray was normal. Barium swallow demonstrated significant gastroparesis thus solidifying the diagnosis of Sjögren’s disease. She had a trial of metoclopramide to improve gastric motility, which was not effective. Following a consultation with Gastroenterologist she had a trial of erythromycin which greatly improved her abdominal symptoms and swallowing. She remains well.

**Case report - Discussion:**

pSS is a chronic systemic autoimmune disease which primarily affects the exocrine glands, most commonly the salivary and lacrimal glands. The prevalence of pSS is estimated to be about 7 per 100,000 person-years. It is characterised by a high female-to-male ratio of 9:1 and the mean age of onset is around the 4th to 5th decade of life.

Signs of an autonomic nervous system dysfunction involving the gastrointestinal and the urinary systems can be observed in the majority of pSS patients. However, this high occurrence is rarely associated with clinically significant symptoms, according to one study. Nevertheless, another study of 38 patients with pSS, demonstrated a higher prevalence of self-reported symptoms of autonomic parasympathetic dysfunction, such as urinary disorder, and gastroparesis (females only) in comparison to controls.

Our patient’s presentation was unusual due to the early age of onset as well as the fact that it was the swallowing problems that triggered autoimmune investigations. She did not report classic sicca symptoms initially, therefore the index of suspicion of underlying primary Sjögren’s syndrome was low. The most prominent clinical symptom was abdominal pain and bloating as well as a feeling of fullness after meals. Her immunology screen was not typical of Sjögren’s syndrome with negative anti-Ro and anti-La antibodies, negative rheumatoid factor, and normal CRP. The patient does not meet ACR criteria for diagnosis of Sjögren’s syndrome, as there is no tissue diagnosis and lacrimation and salivary tests have not been performed yet. However, the presentation with gastroparesis, sicca symptoms, microcystic changes in salivary glands and raised ESR are highly suggestive of primary Sjögren’s syndrome.

**Case report - Key learning points:**

Rarity of pSS and consequently small numbers of patients recruited in the studies of abdominal disorders in pSS make data interpretation difficult.

Symptoms arise from impaired function of exocrine glands located across the entire gastrointestinal system. The autonomic nervous system (ANS) may also be involved in the disease manifested by various autonomic dysfunction (AD) symptoms. In pSS, the degree of exocrine gland destruction and function often correlate poorly. Since exocrine secretion is modulated by the ANS, impaired secretion could partly be due to interference with nervous signals to the exocrine glands.

There is also an immunological hypothesis of symptoms progression in pSS. Such an association between autoimmunity and GI dysfunction has been suggested previously, as there have been reports of coexisting inflammatory bowel disease and severe dysmotility. There is evidence that patients with pSS have antibodies against muscarinic receptors located in vascular smooth muscle cells, particularly in gastrointestinal and genitourinary systems as well as exocrine gland cells.

Gastroparesis is perhaps better known in association with diabetes, with a prevalence between 30% and 50%. However, in one study involving 28 patients with pSS 43% of patients showed signs of impaired gastric emptying, while 29% fulfilled the criteria for gastroparesis. Another study demonstrated that a pathological delay in gastric emptying was present in 70% of patients with pSS.

By presenting this case report we are hoping to generate a discussion and learn from experience of other centres in management of these patients. Given the early age and childbearing potential of the patient treatment options are limited. There are reports of successful use of intravenous immunoglobulin in patients with pSS which was shown to neutralize antimuscarinic M3 receptor antibodies and may improve symptoms of autonomic dysregulation.

